# Skin transcriptomic and selection signature analyses identify *ASIP* as a key gene in cattle coat color determination

**DOI:** 10.3389/fgene.2025.1577647

**Published:** 2025-04-28

**Authors:** Xin Wang, Longxin Xu, Di Zhou, Yanli Lv, Junda Wu, Yuanfeng Zhao, Mengmeng Ni, Wenzhang Zhou, Kaikai Zhang, Hua Wang, Jipan Zhang

**Affiliations:** ^1^ Institute of Animal Husbandry and Veterinary Medicine, Guizhou Academy of Agricultural Sciences, Guiyang, China; ^2^ Guizhou Provincial Breeding Livestock and Poultry Germplasm Determination Center, Guiyang, China; ^3^ College of Animal Sciences, Guizhou University, Guiyang, China; ^4^ College of Animal Science and Technology, Southwest University, Chongqing, China

**Keywords:** cattle, coat color, ASIP, melanogenesis, mRNA-seq, selection signal

## Abstract

**Objective:**

Coat color is a complex trait and plays an important role in breed identification. However, information regarding genes associated with coat color in cattle is limited, especially at the skin transcriptome level.

**Methods:**

We investigated the differential expressed genes (DEGs) and genomic selection signal underlying the coat color variation between black and brown cattle breeds. A total of 19 cattle (Brangus, Angus, Simmental, and Guanling) were performed skin transcriptome analysis and 262 cattle (Angus and Simmental) were performed whole genome analysis.

**Results:**

Angus cattle (black coat) had a significantly higher melanin content in both their hair and skin compared to that of Simmental and Guanling cattle (brown coat). Transcriptomic analysis identified 14,118 expressed genes, with principal component analysis and hierarchical clustering revealing clear differences between black and brown cattle. DEGs analysis across four pairwise breed comparisons highlighted 343 downregulated and 54 upregulated genes common to all comparisons, with the *ASIP* gene (agouti signaling protein) emerging as a key gene linked to melanogenesis. The *ASIP* expression was several dozen-fold higher in brown cattle than in black cattle, suggesting a crucial role in coat color determination. Path-way enrichment and gene set enrichment analysis (GSEA) identified the “Melanogenesis” pathway as significantly enriched and central to coat color variation. Genes such as *FZD10*, *WNT6*, and *ASIP* showed differential expression patterns that correlated with coat color. Genomic analysis revealed strong selection signals in the *ASIP* gene region, with several SNPs exhibiting high linkage disequilibrium. Notably, the mutation type was predominant in Simmental cattle, while the reference allele was more common in Angus cattle.

**Conclusion:**

Based on the skin transcriptomic and genomic analyses, we found that *ASIP* was significantly differential expressed between black and brown cattle breeds and under strong positive selection. These findings provide valuable insights into the genetic basis of coat color variation in cattle and highlight the ASIP gene as a critical determinant of this trait.

## 1 Introduction

Coat color is an essential phenotypic trait that is shaped by long-term natural or artificial selection (Cieslak et al., 2011; Ollivier et al., 2013). The classification, quantification, and registration of coat color are important in animal breeding, as it is a distinguishing characteristic in population status, and can be linked to adaptive traits such as environmental tolerance and disease resistance (Farias et al., 2024). There are numerous famous cattle breeds worldwide with colorful coat colors (Wang et al., 2023; Kunene et al., 2022). For instance, black-coated breeds like Angus and Brangus are highly favored in the beef industry due to their market value and uniform appearance (Farias et al., 2024), while brown-coated breeds such as Guanling are valued for their environment adaptability in the Southwest of China (Xu et al., 2024). In cattle breeding, research attention has focused on meat and milk traits (Congiu et al., 2024), and there is limited information on the functional genes of coat color.

The process of melanin deposition (melanogenesis) is governed by melanocytes, which can be classified into eumelanin and pheomelanin (Marks and Seabra, 2001). Eumelanin is responsible for black to brown colors while pheomelanin is responsible for the red to yellow coloration of mammal coats. Melanogenesis is regulated by a complex network of genes and signaling pathways and several dozens of genes have been linked to coat color in animals (Slominski et al., 2012; Park et al., 2018). Of all mammals, the coat color in horses is the most studied, and genes linked to melanogenesis include *KIT*, *MITF*, *PAX3*, *PATN1*, and *MATP* (McFadden et al., 2024; Mariat et al., 2003). The same genes might have similar pigmentation functions in different species, such as the *MC1R* gene in goats (Li et al., 2019), sheep (Zhou et al., 2023), and water buffalo (Liang et al., 2021). Currently, several genes including *TYR* (Kholijah et al., 2021), *MC1R* (Silva et al., 2021)*, COPA* (Dorshorst et al., 2015), and *KIT* (Jakaria et al., 2023), and seven QTLs (https://www.animalgenome.org/cgi-bin/QTLdb/) were reported to be associated with skin pigmentation in cattle. As a complex trait, coat color in cattle is not fully elucidated.

Fortunately, advances in transcriptomics and genomics now provide powerful tools for identifying the functional genes of complex traits on a molecular level. Messenger RNA sequencing (RNA-seq) is widely used to measure RNA abundance across the whole transcriptome (Stark et al., 2019), and differentially expressed genes (DEGs) can be identified from comparisons. Xiong et al. compared the skin transcriptome data of black and white-coated regions in the same goats, and identified DEGs such as *ASIP*, *DCT,* and *TYRP1* (Xiong et al., 2020); while Zhang et al. identified *TYR*, *TYRP1*, *DCT*, *PMEL*, *MLANA*, and *SLC45A2* as being differentially expressed in black and white sheep, and affecting the pigmentation of the skin and tongue of sheep (Zhang et al., 2024). Leng et al. identified the *DCT*, *TYR*, *TYRP1*, and *MITF* genes involved in melanin pigmentation in embryonic chickens (Leng et al., 2025). Similarly, comparative genomics has been used to identify species-specific genomic regions, aiding in the discovery of functional genes. Based on whole genome sequencing data and the calculated XP-CLR and FST values, Sun et al. identified *IRF2BP2* as a candidate gene affecting fleece traits in sheep (Sun et al., 2024). Also, by calculating the genetic differentiation index (Fst) and nucleotide diversity (theta pi) ratios, Chen et al. identified *ATP5E*, *EDN3*, and *LOC101750371* as candidate genes influencing skin color in black-bone chickens (Chen et al., 2024). Additionally, some studies integrated transcriptome and genomic approaches to enhance statistical power. For example, Tan et al. explored egg production traits in Taihe black-bone silky fowls (Tan et al., 2024), Dorshorst et al. studied coat color in Holstein cattle (Dorshorst et al., 2015), and Ren et al. investigated plumage color in Matahu ducks (Ren et al., 2024).

Encouraged by these successes, we aimed to identify the core genes associated with coat color in cattle on both transcriptomic and genomic levels. Therefore, we collected hair and skin samples from 14 cattle (Angus, n = 6; Simmental, n = 5; Guanling, n = 3) to determine their melanin content and perform transcriptome sequencing. This data was compared to the downloaded skin transcriptome data of five Brangus cattle and the genome data of 262 cattle (Angus, n = 149; Simmental, n = 113) for transcriptomic and genomic analyses. This study conducted a systemical investigation of coat color in cattle from the phenotypical, transcriptomic and genomic levels.

## 2 Materials and methods

### 2.1 Animals and experimental design

**FIGURE 1:**
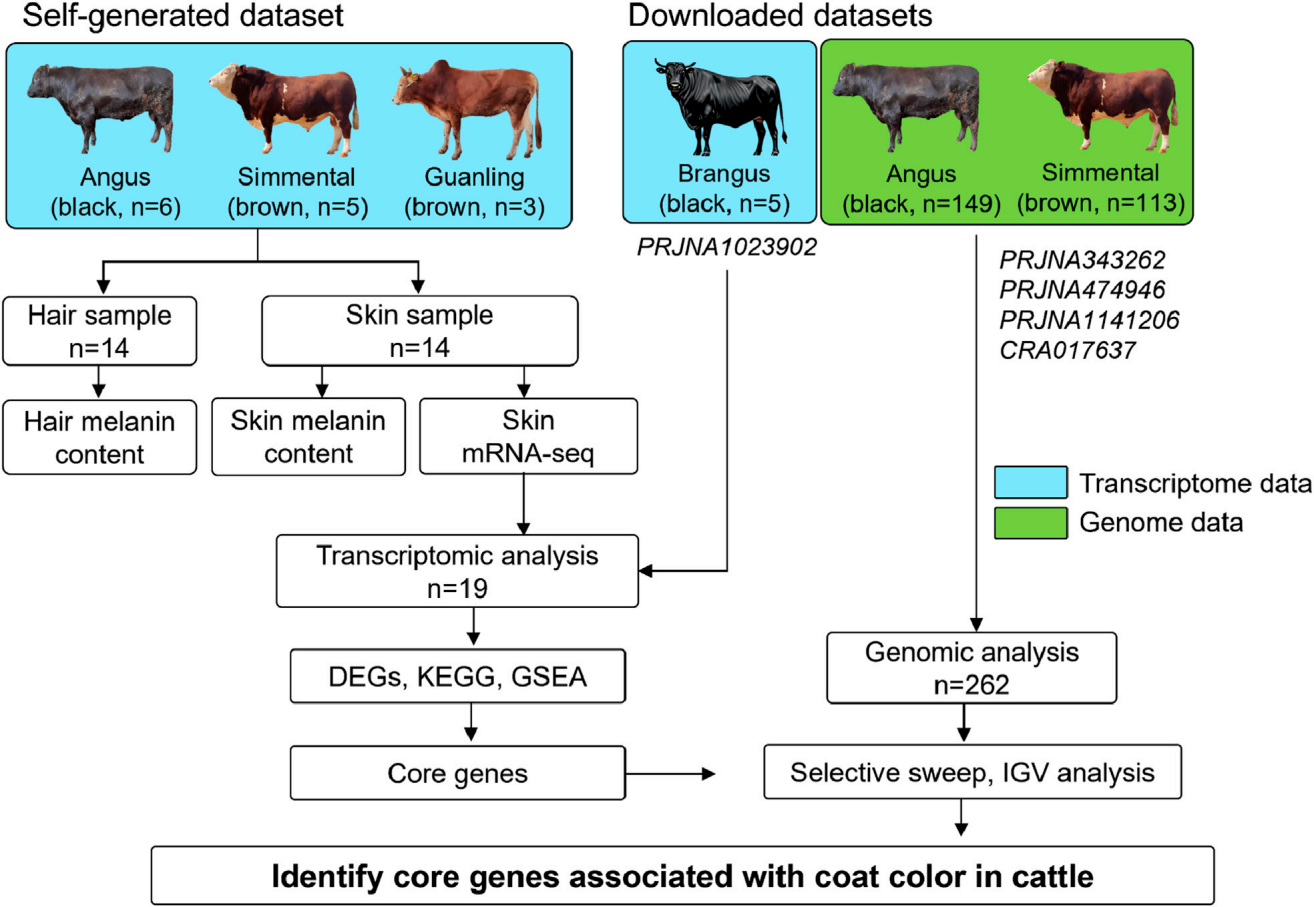
Experimental design for detecting genes associated with coat color in cattle. We selected 14 adult cattle, including Angus (n = 6), Simmental (n = 5), and Guanling (n = 3) for hair and skin sample collection. These samples were used for determining hair/skin melanin content and skin mRNA-seq. Additionally, the publicly available skin transcriptome data of five Brangus cattle and the whole genome data of 149 Angus and 113 Simmental cattle were downloaded. The transcriptome and genome data were used for screening candidate genes related to coat color in cattle.

This study was approved by the Guizhou University Experimental Animal Ethics Committee (No. EAE-GZU-2024-E053). Cattle used in the present study were raised on the Guizhou cattle Industry Group Co., LTD. farm and were fed a diet that met the National Research Council’s requirements for cattle maintenance. These animals were bred by the commercial company, and their purebred status was confirmed based on pedigree records and body characteristics. A total of 14 two-year-old adult animals, including six Angus cattle (coat color: black), five Simmental cattle (coat color: brown), and three Guanling cattle (coat color: brown) were selected and slaughtered in a abattoir. When the animal was pronounced dead, the hair fibers on the body (the center point from the dorsal to abdomen) were shaved and collected to determine hair melanin content. Then, four skin samples of approximately 1 cm × 1 cm were taken from the same site using a surgical scissor. For each individual, two skin samples were stored in a 4% paraformaldehyde fixation solution and two skin samples were snap-frozen in liquid nitrogen, then transferred to the laboratory and stored at −80°C. As shown in Figure 1, 14 hair samples were used for hair melanin content analysis, and 14 skin samples were used for skin melanin content determination and mRNA sequencing.

Additionally, the skin transcriptome data of five Brangus cattle (a breed developed by crossing Angus (*Bos taurus*) and Brahman (*Bos indicus*), and black coat is their typical characteristic) was obtained from the biological project PRJNA1023902 (Álvarez Cecco et al., 2024); and the genome resequencing data of 262 cattle were downloaded from public biological projects, including PRJNA343262 (100 Angus cattle and 40 Simmental cattle), PRJNA474946 (30 Angus cattle and 54 Simmental cattle), and PRJNA1141206 (9 Angus cattle and 9 Simmental cattle), and CRA017637 (10 Angus cattle and 10 Simmental cattle). Detailed information on downloaded transcriptome and genome data is available in Supplementary Tables S1, S2, respectively. Overall, transcriptome analysis is the first step to screen genes for coat color, while genomic analysis is used to observe genomic differences of candidate genes among different coat color breeds. Only genes that show positive results in both transcriptome and genome levels are considered key genes affecting cattle coat color.

### 2.2 Hair and skin melanin content

**FIGURE 2:**
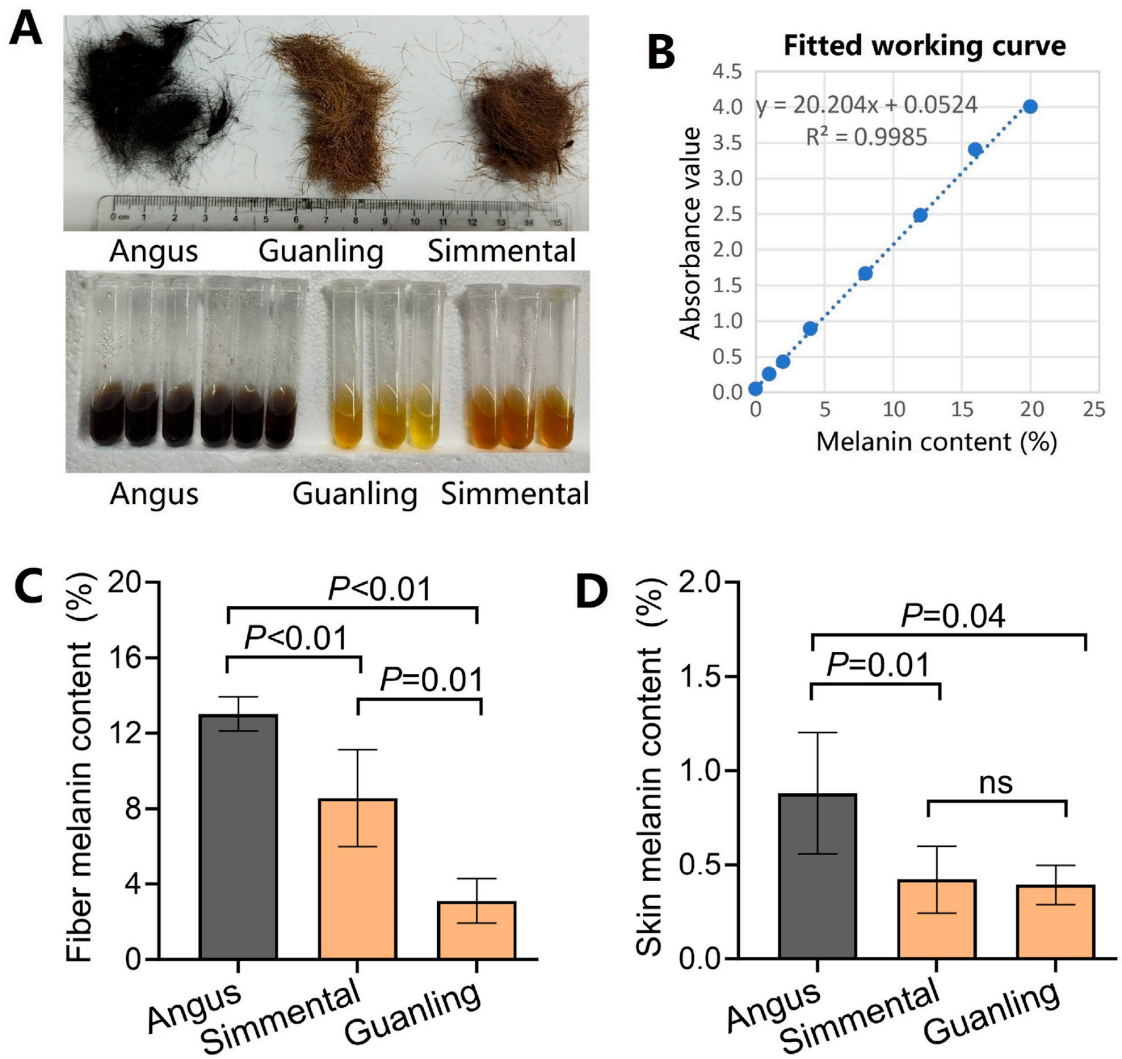
Comparison of hair/skin melanin content in different cattle breeds. **(A)** Photos of the three cattle breeds used in this study. Angus has black coats, while Simmental and Guanling cattle have brown coats. **(B)** The fitted work curve of absorbance corresponds to different concentrations of standard melanin. **(C)** The hair melanin content in Angus, Simmental, and Guanling. **(D)** The skin melanin content in Angus, Simmental, and Guanling.

Coat color was quantified as hair melanin content and skin melanin content based on the NaOH assay method. Hair samples were washed with ethanol, dried, and cut into 1–2-mm lengths. Then, 20–30 mg of each hair sample was placed in a 5 mL centrifuge tube containing 3 mL of 1 mol/L NaOH and water bathed at 95°C for 1 h (Figure 2A). Skin samples were cut into 50–100 mg pieces and placed in a 10 mL centrifuge tube containing 7 mL of 1 mol/L NaOH and water bathed at 95°C for 1 h. The supernatant was removed, and the absorbance was measured at 450 nm using a microplate reader (Bio-rad, America). The working curve was established based on standard melanin (Aladdin, China) at different concentrations, including 0, 0.01, 0.02, 0.04, 0.08, 0.12, 0.16, and 0.2 mg/mL (Figure 2B). Then, the hair melanin content and skin melanin content were calculated using the optical density value and the standard working curve.

### 2.3 Transcriptome sequencing and gene quantization

Fourteen skin samples were sent to Biomarker Technologies Co., Ltd. (Biomarker, Beijing, China) for mRNA extraction, library construction, and mRNA sequencing on the BGI-T7 platform. Adapter sequences, low-quality reads, and those with poly-N’s were removed using the Trimmomatic software (Bolger et al., 2014). Then, the Q20, Q30, GC-content, and sequence duplication levels of the clean data were calculated. The STAR software (Dobin et al., 2013) was used to map clean reads to the reference genome (ARS-UCD2.0 (Bickhart et al., 2017)) and the StringTie (Pertea et al., 2016) software was used to quantify the mRNA expression levels as fragments per kilobase million (FPKM).

### 2.4 Differentially expressed gene (DEG) analysis

This study included four cattle breeds with black and two breeds with brown coats. Principal component analysis (PCA) was used to visualize distance matrices and evaluate the global differences between samples. The hierarchical clustering of the top 5000 genes with the largest variance was generated on a heatmap to reflect the relationship between samples. The DEGs were identified between any two cattle with different coat colors, and four comparisons, including Brangus vs. Simmental, Brangus vs. Guanling, Angus vs. Simmental, and Angus vs. Guanling were generated. For each comparison, we conducted an independent samples t-test (two-tailed, assuming equal variances) for each gene using the R language. Considering that the *P*-values of some comparison groups are relatively high, strict *P*-value correction would lead to an increase in false negatives. Therefore, we did not adjust these *P*-values for all four comparisons. The criteria for identifying DEGs included a *P*-value of <0.05 and fold change (FC) of >2. A Venn diagram was used to visualize overlapping DEGs to find stable DEGs among the above-mentioned comparisons. Stable DEGs that are associated with terms such as “melanin,” “melanogenesis,” or “melanosome” in the following gene annotation, were highlighted in the Venn plot.

### 2.5 Enrichment analysis and gene set enrichment analysis (GSEA)

To identify significant biological signaling pathways, DEGs were annotated by Gene Ontology (GO) analysis and enriched by Kyoto Encyclopedia of Genes and Genomes (KEGG) analysis. The gene annotation was performed and visualized on the OmicShare platform (http://www.omicshare.com/tools, an online platform for data analysis and visualization). Furthermore, the GSEA (Subramanian et al., 2005) tool on the OmicShare platform was used to identify whether there was significant up- or downregulation of melanogenesis-related pathways between black and brown cattle. The GSEA was carried out with Signal2Noise values for all detected 14,118 genes as the ranking metric and the gene expression profile in the core pathway was explored.

### 2.6 Genome data and variant calling

The downloaded genome data of 262 cattle underwent quality control using the Fastp tool [v0.23.4, (Chen et al., 2018)]. Sequence alignment and variant detection were then performed using the Sentieon Genomics software [v202308, (Freed et al., 2017)], where the clean reads were aligned to the cattle reference genome [ARS-UCD2.0, (Bickhart et al., 2017)] using the bwa software [v0.7.17, (Li and Durbin, 2009)], and the BAM files were sorted and duplicates were marked (v2.25). The Sentieon haplotyper module was used to call variants for each sample, generating a genomic Variant Call Format (gVCF) file for each while the joint variant calling was carried out by the Sentieon GVCFtyper module to create a common VCF file. The SelectVariants module in GATK [v4.1.8.1, (McKenna et al., 2010)] was used to split the SNP variants.

### 2.7 Selective sweep and integrative genomics viewer (IGV) analysis

In this part, we aimed to identify the genomic regions that differ between black and brown cattle breeds, and we focused on the genomic regions where the core genes identified in the transcriptome analysis are located. We estimated the signal scanning regions using the fixation index (FST) with VCFtools, employing 10 kb sliding windows and 5 kb sliding steps. The FST values in the top 5% were taken to indicate significant selection. Additionally, Tajima’s D (Sun et al., 2024) statistic was employed to identify potential regional differences between the two cattle breeds. Given the numerous differences between the two breeds, we only conducted detection on the genomic regions around DEGs identified by the transcriptome analysis. The FST and Tajima’s D values on the *ASIP* gene region were focused, and the Linkage disequilibrium (LD) analysis was performed using Haploview [version: 4.2, (Barrett et al., 2005)]. Based on the 19 BAM files of four cattle breeds and the joint VCF file of 262 cattle (Angus, n = 149; Simmental, n = 113), the Integrative Genomics Viewer [v2.19.1, (Robinson et al., 2011)] was used to visualize transcript abundance and variants in the *ASIP* gene region. Finally, the reference allele and mutation of the *ASIP* gene region were compared between Angus and Simmental populations.

## 3 Results

### 3.1 Hair and skin melanin content

The cattle breeds have different coat hairs, with Angus cattle being black, and Simmental and Guanlling cattle being brown. After quantifying the melanin content, we found that the hair melanin content in Angus was 13.0% ± 0.9%, which was significantly higher (p < 0.01) than that of Simmental and Guanling cattle (Figure 2C). Also, the melanin content of Simmental cattle hair was significantly higher (p < 0.01) than that of Guanling. Similarly, the skin melanin content in Angus was 0.88% ± 0.32%, which was significantly higher (p < 0.05) than that of Simmental and Guanling cattle, but there was no difference between the melanin content in the skin of Simmental (0.42% ± 0.16%) and Guanling cattle (0.39% ± 0.11%) (Figure 2D).

### 3.2 Transcriptomic profiles of cattle skin

We obtained 103.4 Gb of raw data from the skin transcriptome sequencing data of 14 cattle (Table 1). On average, each sample was sequenced using approximately 24.7 million reads, and the Q20 (sequencing error rate < 0.01) and Q30 (sequencing error rate < 0.001) were 99.8% and 98.7%, respectively. The downloaded skin transcriptome data of the five Brangus cattle (Supplementary Table S1) were included in the analysis. Then, quality control, reads mapping, transcript assembly, and gene quantification were performed on the transcriptome data. After removing genes without symbol names, and genes with extremely low expression (average FPKM <0.1), we obtained an FPKM matrix with 14,118 genes across 19 samples (Supplementary Table S3). All the following analyses were performed on the FPKM matrix with high-quality data.

**TABLE 1:** The transcriptome dataset of 14 cattle skin samples sequenced by this study.

No.	Sample	Breed	Coat color	Reads	Base (Gb)	Q20 (%)	Q30 (%)	GC (%)	Mapping rate (%)	Skin melanin content (%)	Hair melanin content (%)
1	Ag1	Angus	Black	24918250	7.46	99.88	99.12	47.63	90.62	13.06	0.90
2	Ag2	Angus	Black	23886023	7.14	99.6	98.28	51.39	94.09	13.26	1.47
3	Ag3	Angus	Black	28268735	8.46	99.55	98.06	49.33	94.12	13.56	0.72
4	Ag4	Angus	Black	22562245	6.74	99.91	99.17	48.71	95.35	12.02	0.63
5	Ag5	Angus	Black	27700948	8.29	99.58	98.17	50.41	91.64	14.26	0.95
6	Ag6	Angus	Black	28418872	8.51	99.59	98.26	50.17	88.73	11.95	0.61
7	Gl1	Guanling	Brown	23675752	7.08	99.91	99.1	45.15	79.41	2.05	0.45
8	Gl2	Guanling	Brown	20648245	6.17	99.55	98.17	49.36	90.03	2.89	0.27
9	Gl3	Guanling	Brown	26203738	7.84	99.83	98.7	48.69	96.93	4.36	0.45
10	St1	Simmental	Brown	23671715	7.08	99.9	99.13	48.84	96.45	10.07	0.35
11	St2	Simmental	Brown	21585320	6.45	99.88	99.13	48.76	94.26	10.56	0.36
12	St3	Simmental	Brown	28652627	8.56	99.87	99.08	48.19	86.51	10.19	0.64
13	St4	Simmental	Brown	21533108	6.44	99.61	98.34	50.84	96.39	4.52	0.57
14	St5	Simmental	Brown	23940084	7.16	99.89	99.05	49.68	96.98	7.48	0.19

### 3.3 Differentially expressed genes between black and brown cattle

**FIGURE 3:**
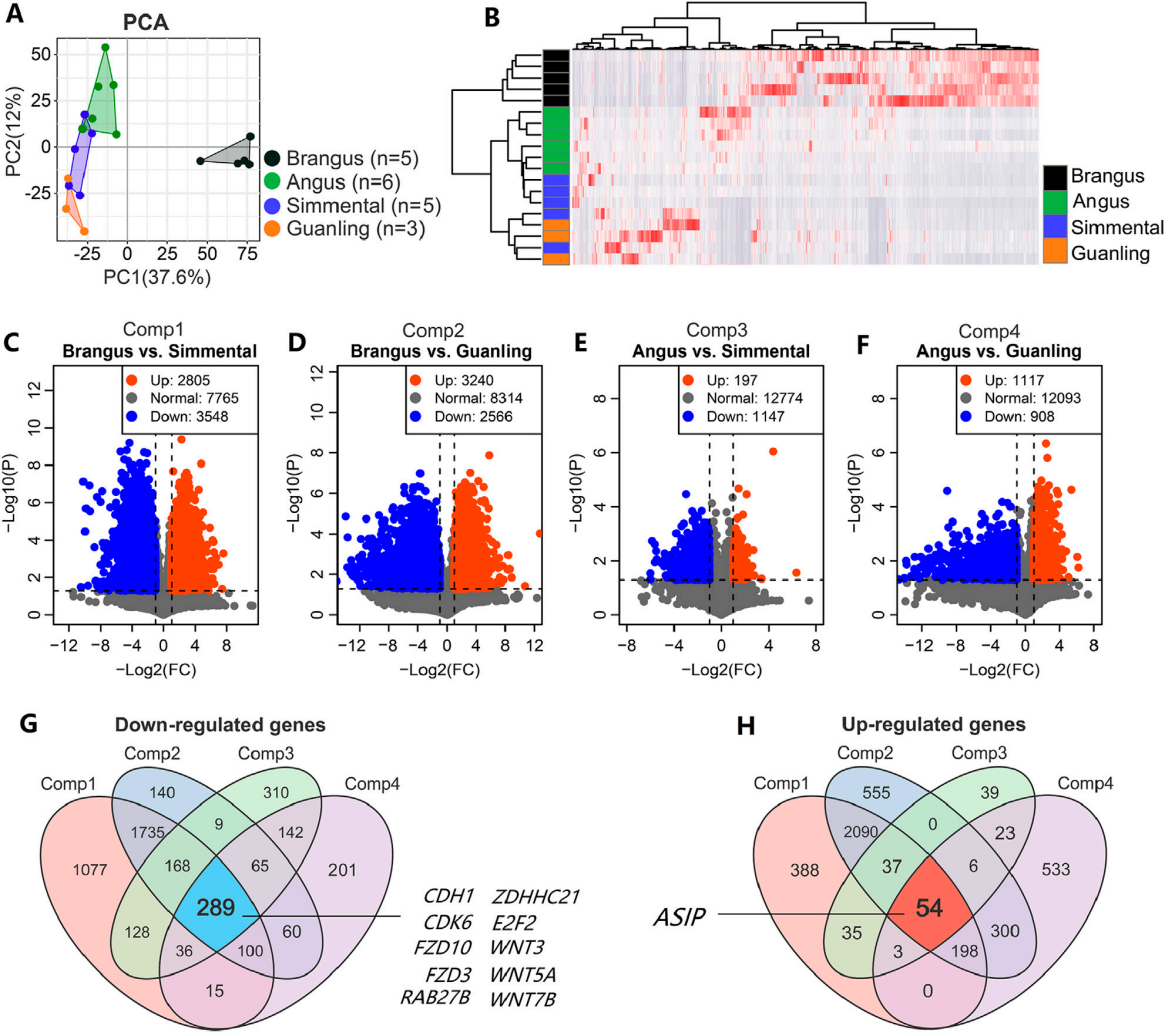
Core genes identified based on transcriptome data of cattle with different coat colors. **(A)** The PCA score plot of 19 cattle based on transcriptome data showing that overall difference among animal groups was significant; **(B)** Hierarchical clustering heatmap of the top 5000 genes with the largest variance across the 19 samples. Serial volcano plots displaying DEGs between black and brown cattle, including **(C)** Brangus vs. Simmental, **(D)** Brangus vs. Simmental, **(E)** Angus vs. Simmental, and **(F)** Angus vs. Guanling. The Venn diagram for screening for stable significant down- **(G)** and upregulated **(H)** genes is based on these four comparisons.

The PCA plot (Figure 3A) and heatmap (Figure 3B) of the top 5000 highly variable genes displayed the overall differences between cattle breeds, with higher repeatability within the intra-group samples than among the inter-group samples. By comparing any two breeds with different coat colors, we identified DEGs related to this trait. For example, 2805 upregulated and 3548 downregulated DEGs were identified between Brangus and Simmental (Figure 3C), while 3240 up- and 2566 downregulated DEGs were identified between Brangus and Guanling (Figure 3D). There were 197 up- and 1147 downregulated DEGs identified between Angus and Simmental (Figure 3E), and 1117 up- and 908 downregulated DEGs were identified between Angus and Guanling (Figure 3F). The significantly down- and upregulated genes of these four comparisons can be found in Supplementary Table S4. Through a Venn analysis, 289 downregulated genes (Figure 3G) were found to overlap in four comparisons. Of those, genes including *CDH1*, *FZD10*, *FZD3*, *GPR143*, *WNT3*, *WNT5A*, and *WNT7B* were associated with coat color in animals. Meanwhile, 54 upregulated genes (Figure 3H) overlapped these four comparisons, with the *ASIP* gene being the unique gene related to melanogenesis. In DEGs analysis, we did not correct the P-values because we found very few DEGs were obtained in comparisons of “Angus vs. Simmental” and “Angus vs. Guanling” after Benjamini-Hochberg or Bonferroni corrections (Supplementary Tables S5–S7). Interestingly, even after strict P-value correction, the *ASIP* gene still showed robust differential expression between black and brown breeds (Supplementary Figure S1).

### 3.4 The significant pathways and core genes related to coat color

**FIGURE 4:**
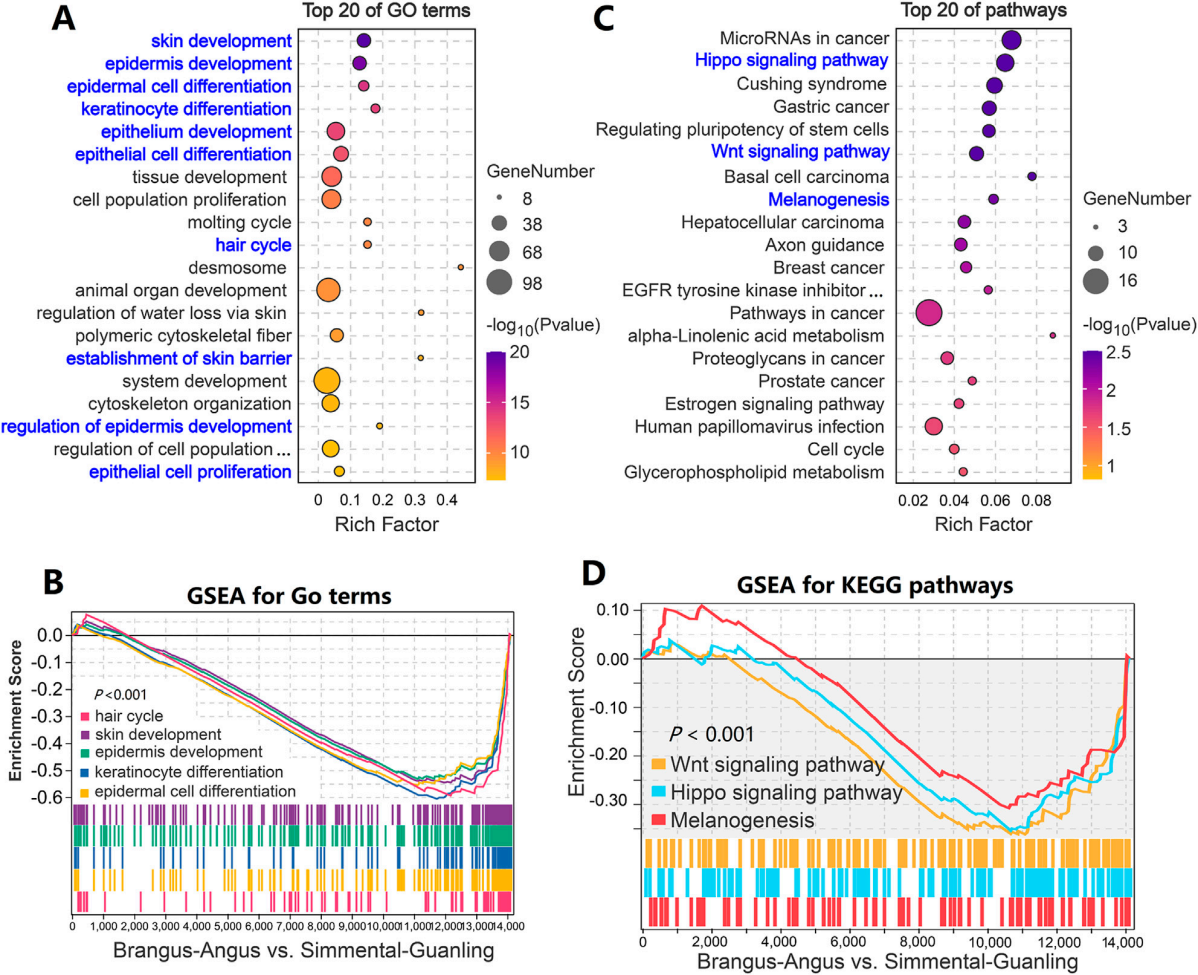
Function enrichment analysis of differentially expressed genes in cattle with different coat colors. **(A)** GO term enrichment analysis based on 343 overlapped genes. **(B)** GSEA analysis showed that genes from five GO terms decreased significantly overall. **(C)** KEGG enrichment analysis based on 343 overlapping genes. **(D)** GSEA analysis showed that genes from three pathways decreased significantly overall.

We performed the enrichment analysis to identify significant GO terms and biological pathways on the overlapping genes dataset, including 289 down- and 54 upregulated genes. As shown in Figure 4A, many terms significantly related to skin biology, such as “skin development,” “epidermis development,” “epidermal cell differentiation”, and “keratinocyte differentiation” were identified. These GO terms were significantly down-regulated (*p* < 0.001) from the black to the brown cattle group (Figure 4B). Within the top 20 KEGG pathways, three pathways including the “Hippo signaling pathway,” “Wnt signaling pathway,” and “Melanogenesis” that related to hair and skin biology were significantly enriched (*p* < 0.01, Figure 4C; Supplementary Table S8). The GSEA results showed that the “Hippo signaling pathway,” “Wnt signaling pathway,” and “Melanogenesis” pathways were significantly downregulated (*p* < 0.001) from the black to the brown cattle group (Figure 4D; Supplementary Table S9).

**FIGURE 5:**
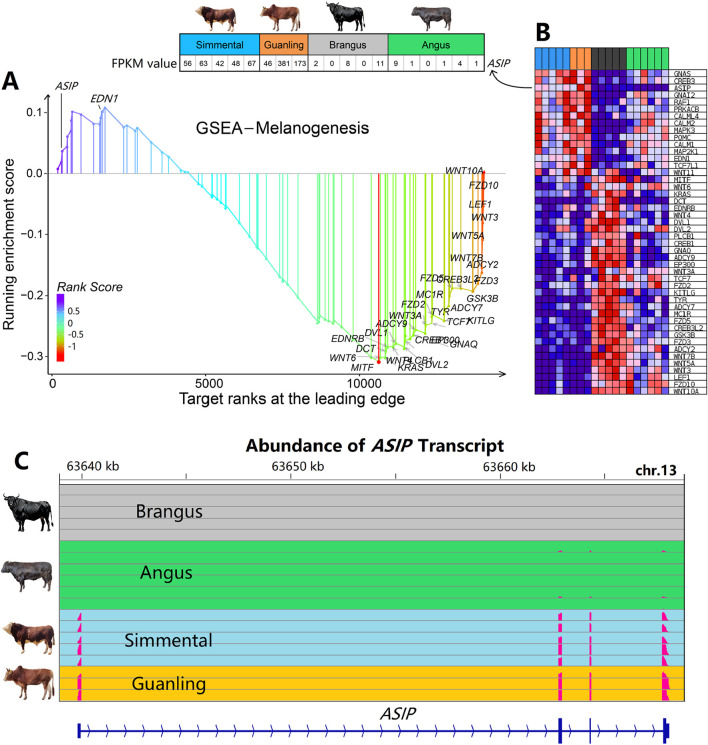
Gene set enrichment analysis and gene profile of the “Melanogenesis” pathway in cattle with different coat colors. **(A)** GSEA identified the “Melanogenesis” signaling pathway as significant (P < 0.05). **(B)** Expression profile of genes that belong to the “Melanogenesis” pathway. The expression of the *ASIP* gene is several dozen-fold higher in brown cattle than in black cattle, showing the highest consistency within cattle of the same coat color. **(C)** Abundance of *ASIP* transcripts across four cattle breeds. Expression is notably higher in Simmental and Guanling cattle compared to Brangus and Angus.

“Melanogenesis” is a well-known biological pathway that determines the pigment and coat color in animals. As shown in Figure 5A, most genes in the “Melanogenesis” pathway decreased in expression levels, such as *FZD2*, *FZD10A*, and *WNT6*; while few genes increased in expression, such as *ASIP* and *EDN1*. According to Figure 5B, results showed that genes on this pathway were expressed differentially in Brangus and Angus cattle. However, the *ASIP* gene presented the highest consistency in cattle with the same coat color, while the expression level of the *ASIP* gene in brown cattle was several dozen-fold higher than that in black cattle. As shown in Figure 5C, the transcript was detected to have a high abundance in brown cattle (both Simmental and Guanling) while almost no abundance was detected in black cattle (both Brangus and Angus).

### 3.5 Selection signal and variants of the ASIP gene

**FIGURE 6:**
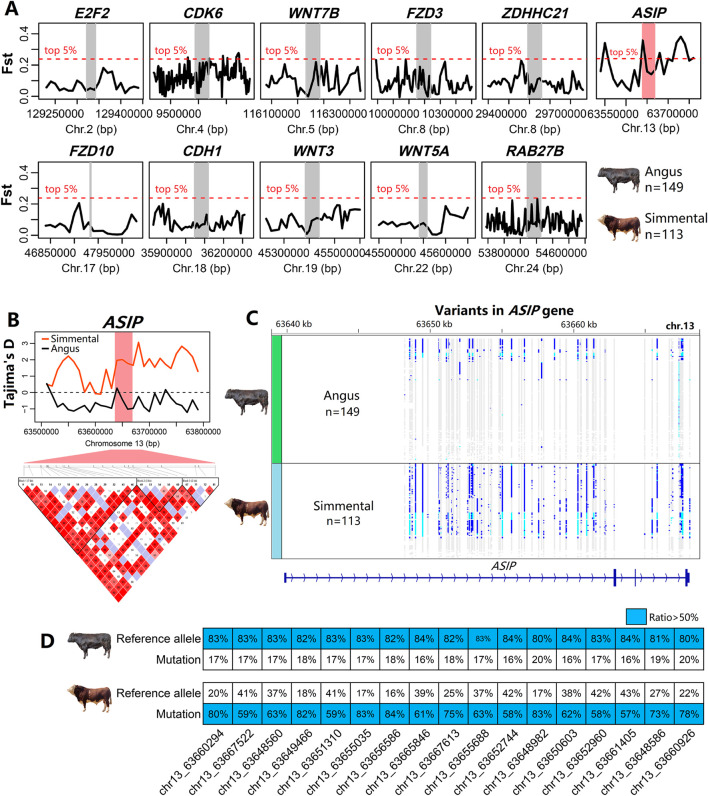
Selection signals of genomic regions around 11 DEGs in cattle. **(A)** Among these DEGs, only the genomic regoin that *ASIP* was located shows a significant peak in Fst analysis (top 5%), indicating strong genetic differentiation. **(B)** The Tajima’s D analysis suggested possible selective pressure affecting the region that *ASIP* was located. Linkage disequilibrium (LD) plot showed that SNPs around the *ASIP* locus were tightly linkaged. **(C)** Genetic variant distribution for the *ASIP* gene demonstrates contrasting SNP profiles between Angus and Simmental cattle. **(D)** A comparison of SNP allele frequencies shows that, for 17 SNPs within the *ASIP* gene, Simmental cattle predominantly have the mutation type, whereas Angus cattle mostly have the reference allele.

Based on the genomic data of Angus (n = 149) and Simmental (n = 113) cattle, we performed the selection signal analysis. Of these 11 DEGs, only the windows where the *ASIP* genes were located were under strong selection (Figure 6A). This signal of selection was also supported by the and Tajima’s D statistics (Figure 6B). Besides, several dozen SNPs in the *ASIP* gene had strong linkage disequilibrium). The gene length of *ASIP* was 28.3 Kb, and it had four exons. Figures 6C,D shows the reference allele and mutation in the *ASIP* gene. Most of the Simmental cattle had the mutation type of the SNPs and for the 17 SNPs (Supplementary Table S10) in the *ASIP* gene, the reference allele was prominent in Angus cattle, while the mutation type was prominent in Simmental cattle. These genotypes may result in the differential expression of *ASIP*, thereby affecting the coat color of cattle.

## 4 Discussion

This study conducted a comprehensive investigation into coat color from phenotypic, transcriptomic, and genomic perspectives, emphasizing the pivotal role of the *ASIP* gene in determining coat color in cattle. Among coat color genes, *ASIP* exhibited the highest within-group consistency and the greatest between-group differences at the transcriptome level, and it was under the strongest selective pressure between different coat color breeds at the genome level. Our study highlights the significant role of the *ASIP* gene in determining cattle coat color.

Coat color is a complex trait, although easily visible to the naked eye. It presents on the outside as coat color, but as melanin deposition internally and plays an important role in the intersection of evolution, genetics, and developmental biology (Hoekstra, 2006). Few studies have determined the hair and skin melanin content of vertebrates, although they are good quantitative indicators of the degree of melanin deposition. The NaOH method for measuring hair and skin melanin content has proven effective in distinguishing between black and brown coat cattle. However, the precision of this method may be limited because it relies on cumbersome experimental procedures and absorbance measurements that are prone to interference.

As the largest livestock worldwide, the coat color of cattle has attracted a lot of interest. Beyond the *ASIP* gene, other famous color-related genes such as *MC1R* (Silva et al., 2021; Mohanty et al., 2008), *KIT* (Jakaria et al., 2023; Fontanesi et al., 2010), *TYR* (Kholijah et al., 2021; Schmutz et al., 2004), and *MITF* (Philipp et al., 2011), have also been associated with coat color in cattle. However, these genes did not show significant differential expression in the present study. The expression of these genes was unstable in different varieties, and the combined analysis of multiple breeds did not show a low statistical probability value. The regulatory mechanisms of coat color may be different in cattle breeds, so the Venn analysis of these four comparisons (any two of black and brown cattle) did not always identify these other color-related genes except the *ASIP* gene.

Evidence supporting the effect of *ASIP* on the color of cattle hair was obtained not only from transcriptional expression (Albrecht et al., 2012; Girardot et al., 2006) but also from the genome. Trigo et al. found that the structure variant of the *ASIP* sequence causes darker coat pigmentation in white-coated Nellore (Trigo et al., 2021) and Zebu cattle (Trigo et al., 2023). Additionally, the following reports showed that the genomic region of the *ASIP* gene has been subjected to strong selective pressures across various cattle breeds: (1) Xu et al. analyzed Holstein, Angus, Charolais, and Brahman cattle (Xu et al., 2015); (2) Bertolini et al. focused on Reggiana and Modenese cattle (Bertolini et al., 2022); (3) Rajawat et al. examined several Indian cattle breeds (Rajawat et al., 2023); (4) Mustafa et al. studied Pakistani cattle breeds (Mustafa et al., 2018); and (5) Guan et al. studied Chinese native cattle breeds (Guan et al., 2022). In the present study, a comparative analysis of genome resequencing data from 149 Angus and 113 Simmental cattle revealed significant differences in mutation sites within the *ASIP* gene region between the two breeds. Black Angus cattle predominantly carried the reference genotypes, whereas brown Simmental cattle mainly had mutant genotypes. However, neither breed showed complete fixation of these genotypes. This discrepancy could be attributed to two factors: (1) potential errors in sample records or (2) the global distribution of Simmental and Angus cattle, which may have resulted in populations that are not entirely purebred.

The skin transcriptome data of five Brangus cattle used in the study were downloaded from the PRJNA1023902 project (Álvarez Cecco et al., 2024), which also included a heat-stressed group of Brangus cattle. However, we retained the normal group while the heat-stressed group was excluded because we found that *ASIP* might be affected by heat-stress treatment. Specifically, the expression levels of *ASIP* in the heat-stress group exhibited differential expression compared to the normal group (*P* = 0.06, t-test based on two-tailed and equal variance; data not shown). In the original project from which we downloaded the skin transcriptome data, some animals were recorded as “red”. In fact, although black is the classic coat color of Brangus cattle, red individuals also exist. Since the original authors did not provide individual images, it is difficult to quantify the differences in color intensity among black and red individuals. Therefore, individuals belonging to Brangus were treated as a single experimental group. In this study, based on multi-breed skin transcriptome comparisons and genomic evidence, the significant role of *ASIP* in determining cattle coat color was emphasized. We should recognize that the study conclusions are limited by the experimental materials used and the analytical strategies employed.

As the above-mentioned, coat color genes may exhibit dynamic expression in response to heat stress conditions to regulate body temperature. By comparing hair cortisol and serotonin levels in lactating Holstein cows with different coat colors under heat stress conditions, Ghassemi et al. pointed out that white coats are preferable for dairy cows to cope with thermal stress (Ghassemi et al., 2017). Melanin synthesis and deposition in hair and skin primarily serve to absorb heat energy for maintaining body heat balance (Leite et al., 2020; Al-Ramamneh and Gerken, 2024). Under heat stress, the demand for melanin synthesis decreases, leading to a reduction in the activity of the melanogenesis pathway at tissue and cellular levels. Therefore, the changes in the expression levels of *ASIP* reflect the organism’s adaptive response to thermal stress.

Importantly, *ASIP* is not only expressed in the skin but also widely in other tissues, including adipose, heart, liver, kidney, and ovary (Albrecht et al., 2012; Girardot et al., 2006). Besides its role in melanogenesis, *ASIP* has been linked to fat deposition traits, oocyte maturation in the ovary, and lipid composition in the mammary gland. Specifically, *ASIP* plays an important functional role in promoting oocyte maturation and subsequent embryonic development in cattle (Chaney et al., 2024). Xie et al. performed an *ASIP* gene knockout study in bovine mammary epithelial cells using CRISPR/Cas9 technology and determined its significant role in regulating lipid metabolism and fatty acid composition (Xie et al., 2022). Liu et al. found that genomic variants (one indel and three SNPs) in introns of the *ASIP* gene were significantly correlated with backfat thickness in cattle (Liu et al., 2019) and Fernandes et al. also found that *ASIP* was associated with backfat thickness in cattle (Fernandes et al., 2016). Within muscle tissue, the ASIP protein is released by adipocytes and potentially functions as a signaling molecule facilitating communication between intramuscular fat and muscle fibers (Liu et al., 2018). Thus, in addition to its primary role in coat color, *ASIP* also plays significant biological roles in various tissues in cattle.

## 5 Conclusion

This study investigated the coat color in cattle from phenotypical, transcriptomic, and genomic levels to determine the core genes responsible for coat color variation in Brangus, Angus, Simmental, and Guanling cattle. The hair and skin melanin content were significantly difference between black-coated (Brangus and Angus) and brown-coated (Simmental and Guanling) cattle. Based on the skin transcriptome data of 19 cattle, we identified the *ASIP* gene that was significantly differentially expressed between black and brown cattle groups. Based on the selection signal and integrated genomic viewer analyses, we found that *ASIP* was under strong positive selection between Angus and Simmental cattle. These findings provide further evidence to deepen our understanding of coat color in cattle from phenotypical, transcriptomic, and genomic levels.

## Data Availability

The datasets presented in this study can be found in online repositories. The names of the repository/repositories and accession number(s) can be found in the article/Supplementary Material.
