# Supplementary material for: Skin transcriptomic and selection signature analyses identify ASIP as a key gene in cattle coat color determination
Source: Front Genet. 2025 Apr 28;16:1577647. doi: 10.3389/fgene.2025.1577647 (PMC12066557; doi:10.3389/fgene.2025.1577647)
Supplement: Supplementary file 1 [file DataSheet1.pdf]

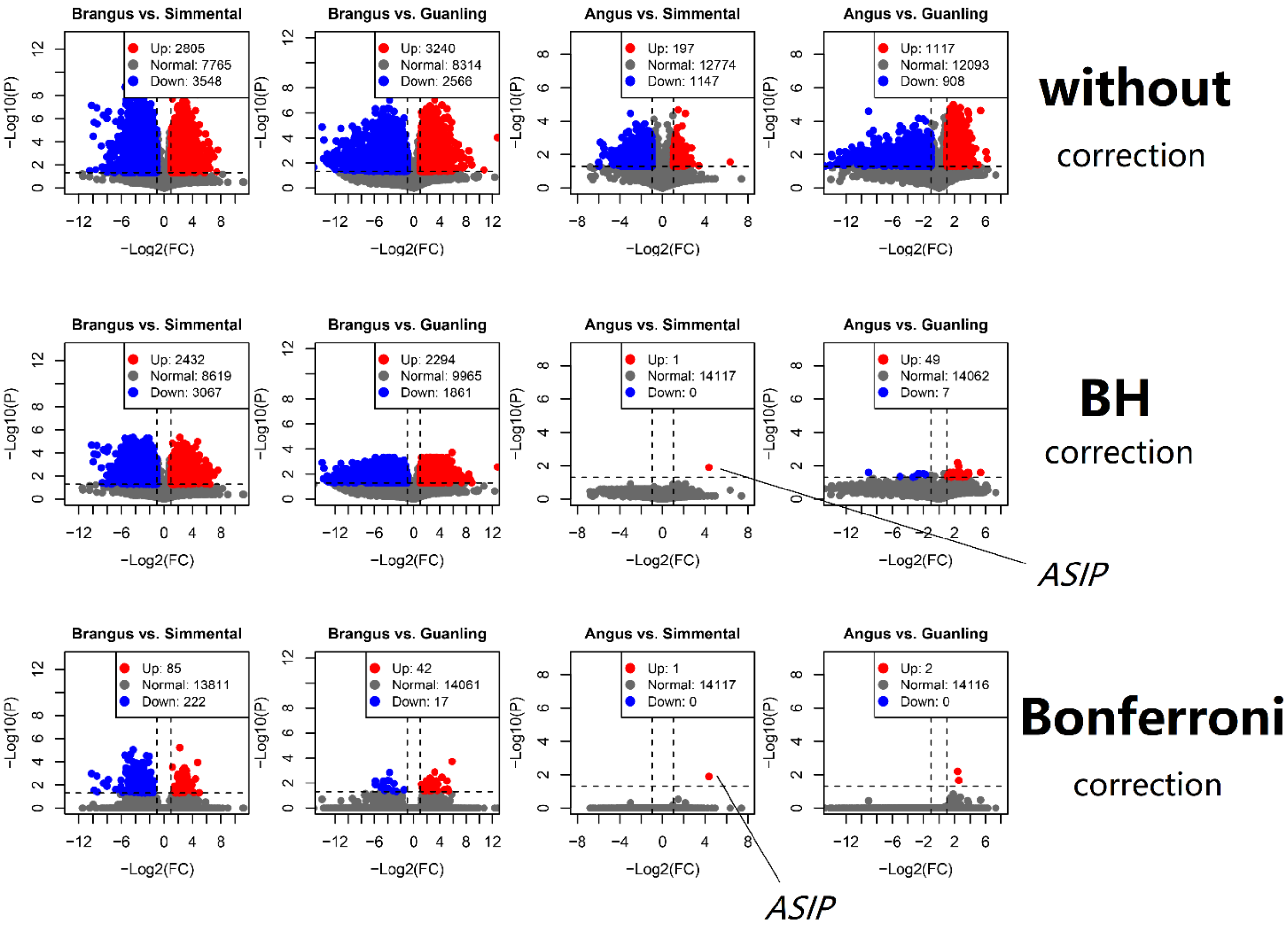

Figure S1: Series of volcano plots for breed comparisons: p-values uncorrected, Benjamini-Hochberg -corrected, and Bonferroni-corrected
